# Mimics and Chameleons in Guillain-Barré Syndrome

**DOI:** 10.7759/cureus.18325

**Published:** 2021-09-27

**Authors:** Fadila ., Praveen Kumar, Md Faraz Omair

**Affiliations:** 1 Institute of Child Health, Sir Ganga Ram Hospital, New Delhi, IND; 2 Division of Pediatric Neurology, Institute of Child Health, Sir Ganga Ram Hospital, New Delhi, IND; 3 Department of General Medicine, GreenLife hospital, Patna, IND

**Keywords:** guillain barre’s syndrome (gbs), demyelinating dieases, peripheral neuropathies, autoimmune diseases, acute flaccid paralysis

## Abstract

Guillain-Barré syndrome (GBS) is an immune-mediated disease of the peripheral nervous system that is triggered by both infectious processes and post-immunization conditions. It is, therefore, more prevalent during infectious outbreaks. While the classical clinical presentation of ascending paralysis is easy to recognize, GBS is a heterogeneous entity comprising several variants, atypical presentations, and incomplete forms that may make the diagnosis challenging. Early recognition is key because the disease could be rapidly fatal. Monitoring for progression of illness, fluctuations in vital signs, and prompt initiation of intravenous immunoglobulin are the mainstays of treatment.

## Introduction

Guillain-Barré syndrome (GBS) is an autoimmune disorder that is classically thought of as a post-infectious polyneuropathy, involving mainly motor, but also sensory and sometimes autonomic nerves. First described by Landry in 1859, Georges Guillain and John Barré diagnosed two soldiers with this illness, and also described the key diagnostic abnormalities of albumino-cytological dissociation and ascending paralysis. Since then, the diagnostic criteria for GBS have been developed based on clinical analysis, cerebrospinal fluid (CSF) findings as well as nerve conduction study results [1]. However, the diagnosis of GBS can be challenging, particularly when one encounters its subtypes, variants, and atypical presentations. The frequency of occurrence of these variants is partially related to the geographical area in which the disease is reported; for example, Miller Fisher syndrome accounts for 5% of cases in western Europe with an even higher prevalence reported from Taiwan and Japan [2]. Additionally, Miller Fisher syndrome may, in practice, present with other cranial nerve palsies resulting in a Miller-Fisher-Guillain-Barré overlap syndrome [3], or at times it may present with an isolated ocular nerve palsy. The pharyngeal-brachial variant is another regional form of GBS that could pose a substantial diagnostic challenge. When paresis is restricted to the legs, but with the later involvement of the arms with sensory signs, it is considered a paraplegic variant [4]. Bickerstaff brainstem encephalitis is another variant that can present with acute ataxia and hyper-somnolence. Although this syndrome affects people of all ages and is not a hereditary condition, GBS poses unique challenges in young children who frequently present atypically, and in whom the neurological examination is difficult. Therefore, it is noteworthy that although the diagnosis of GBS is straightforward, it could be challenging in atypical cases, in the setting of severe pain preceding weakness, in young children, and in low-income countries with poor diagnostic facilities. In this case series, we describe five interesting cases of the variants of GBS followed by a brief review of the literature.

## Case presentation

Case 1

A three-year-old previously healthy male child presented to us with a history of fever and headache for the past seven days. For this, he had been given symptomatic treatment at a local clinic. On the third day of fever, the parents had noticed difficulty in vision, which was described as the child not being able to pick up objects and missing his intended target when given something to grab. There had been no tremors noticed, but the parents had observed drooping of both eyelids. On clinical examination, the child was active, alert, and playful; there was bilateral ptosis with restriction of extraocular muscle movements. Pupils were equal-sized and reactive to light. Muscle tone and power were adequate in all limbs, but deep tendon reflexes (DTRs) could not be elicited. Bowel and bladder control was intact. The child’s gait was unsteady, but there were no other signs of cerebellar involvement. There were no signs of meningeal irritation. We performed a contrast-enhanced MRI of the brain, which was reported normal. Nerve conduction velocity (NCV) study done on the first day of admission (seventh day of illness) was also normal. CSF analysis was suggestive of albumino-cytological dissociation (cell count: 2; protein: 62 mg/dl; sugar: normal). GQ1b antibody was sent, which returned negative. At that time, our suspicion for Miller Fisher syndrome was strong based on the ataxic gait, ophthalmoplegia, and absent DTR. The child was treated with intravenous immunoglobulin (IVIG) of 2 gm/kg along with physiotherapy. Over the next four weeks, the child's condition improved, and he was able to walk unsupported; his ptosis improved, and no restriction of movement of extraocular muscles was noticed on the follow-up.

Case 2

A four-year-old boy presented to us with fever, headache, and dull aching abdominal pain for the past seven days. He had started complaining of double vision from the fifth day of illness. He had also experienced difficulty in chewing and swallowing for two days before admission. MRI brain had been done at a local radiology center, which had been reported normal. At the time of presentation to us, the child was drowsy but arousable. There was bilateral lower motor neuron type facial nerve palsy, external ophthalmoplegia, jaw weakness, decreased palatal movements, and a weak gag reflex. Tone and power were age-appropriate in all four limbs. There was an exaggerated bilateral knee reflex. Bowel and bladder control was intact. Truncal ataxia was present along with a broad-based gait. There was no neck rigidity. The child was admitted to our facility on the eighth day of illness. MRI brain with contrast was repeated at our center, which again returned normal. CSF analysis was also normal. NCV was done on the ninth day of illness, which showed distal symmetrical motor axonal involvement of facial nerve bilaterally, abnormal blink reflex with absent conduction along the afferent and efferent pathways of the blink reflex. The overall final clinical impression was of Bickerstaff brainstem encephalitis. The child was treated with IVIG of 2 gm/kg. Following this, there was no further progression of weakness. Sensorium improved after the third day of admission, and the child was discharged after five days of hospital stay.

Case 3

**Figure 1:**
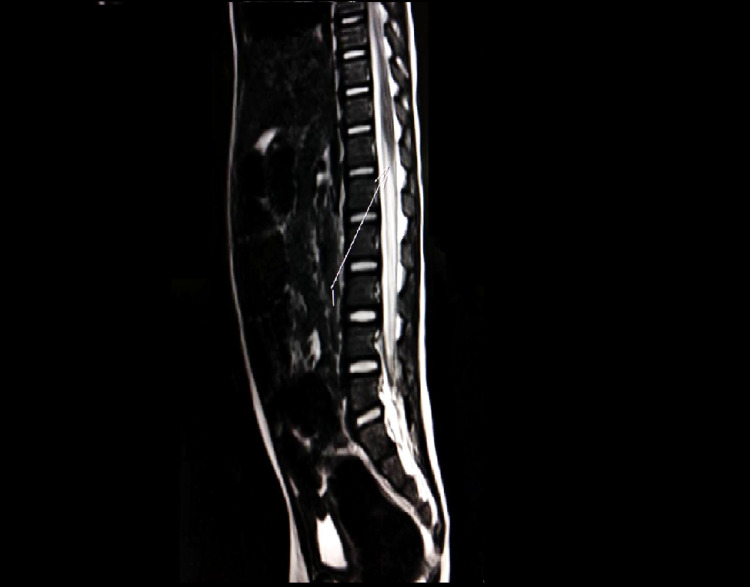
Saggital section MRI spine showing thickened conus medullaris and cauda equina nerve root with intense post-contrast enhancement MRI: magnetic resonance imaging

**Figure 2:**
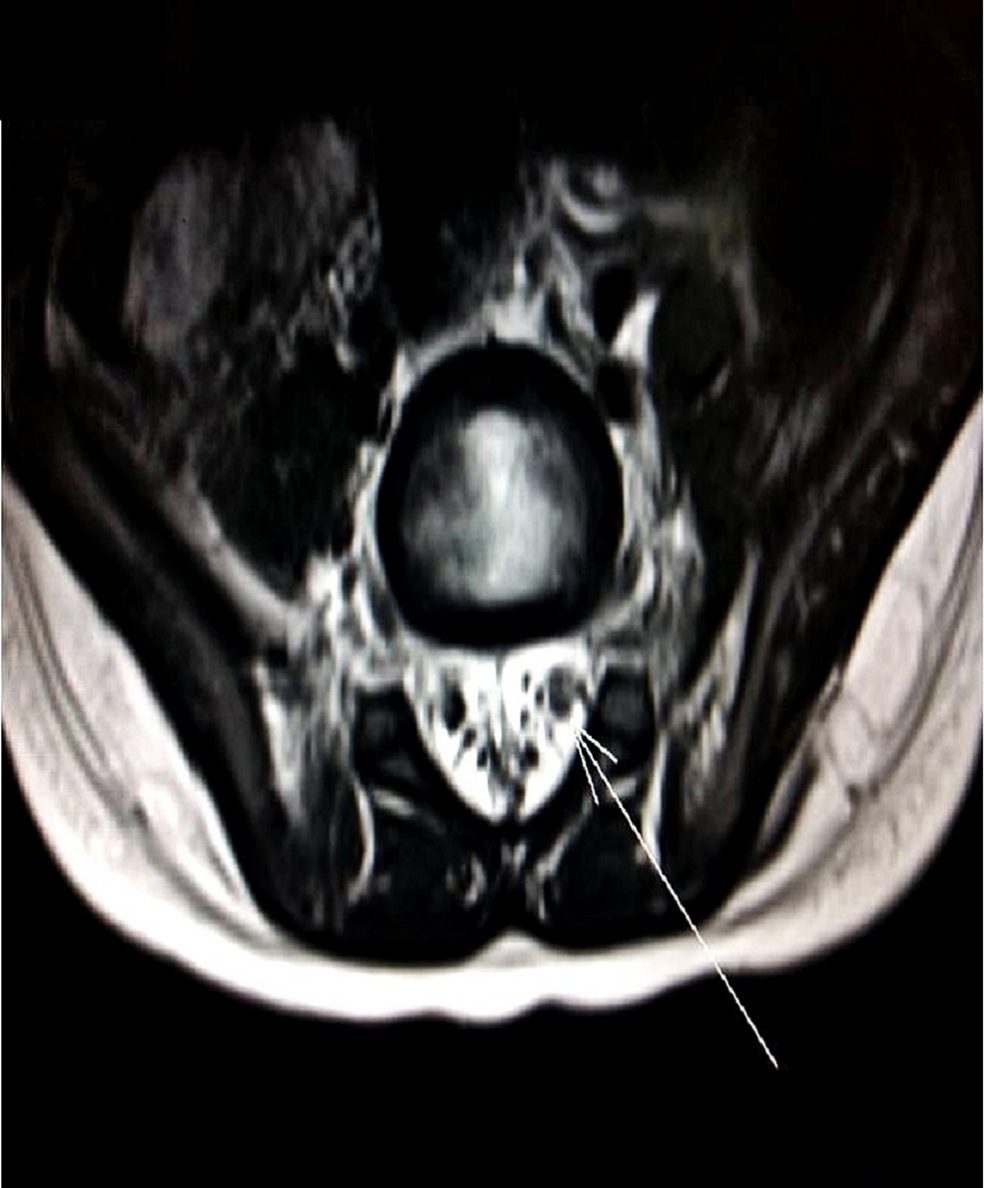
Axial section MRI spine showing thickened nerve root with intense post-contrast enhancement MRI: magnetic resonance imaging

A two-year-old boy presented with fever and progressive weakness of both legs for five days. There was a history of urinary retention for the last four days. In the week preceding the presentation, the child had suffered from an upper respiratory tract infection for which he had been managed symptomatically. On examination, the child was irritable and uncooperative, and no focal neurological deficits were identified. There was hypotonia in all four limbs, along with truncal weakness, and power of 3/5 in all limbs. DTRs were present and plantar reflex was normal. No neck rigidity was noted. We performed an MRI brain, which was normal. However, the MRI spine showed thickened conus medullaris and cauda equine nerve root with intense post-contrast enhancement (Figures 1, 2).

NCV done on the eighth day of sickness was normal. Lumbar puncture showed albumino-cytological dissociation (cell count: 13, protein: 92 mg/dl, sugar: 56 mg/dl). We treated the patient with IVIG under the working diagnosis of GBS variant in light of the urinary retention. Physiotherapy and occupational therapy were done every day. The child was discharged after seven days of admission. His truncal weakness had improved, and he was passing urine normally, and power in the limbs at the time of discharge was 4/5.

Case 4

A two-and-a-half-year-old female child with a history of fever and cough for five days, 10 days before presentation to us, presented with complaints of right upper limb weakness for four days, followed by inability to sit up on her own, and left upper limb weakness. MRI brain with the cervical spine and brachial plexus performed at a local center had been reported normal. CSF analysis had also been done there, which had been within normal limits. The child was then admitted to our hospital. On examination, she was irritable but consolable. Bilateral facial nerve weakness was present. Truncal weakness was also present. Power in the right upper limb was 2/5, and it was 3/5 in the left upper limb. Lower limb power was age-adequate. Biceps and triceps jerk was absent bilaterally. DTR in lower limbs was normal, and bowel and bladder control were intact. There was no neck rigidity. NCV was done at our center, which showed distal symmetrical axonal neuropathy in bilateral upper limbs. We treated the patient as a case of a bi brachial variant of GBS. Following IVIG administration of 2 gm/kg over 72 hours and physiotherapy, the child was able to sit unsupported and had improved movement in both upper limbs. She was discharged after seven days of hospital stay.

Case 5

**Figure 3:**
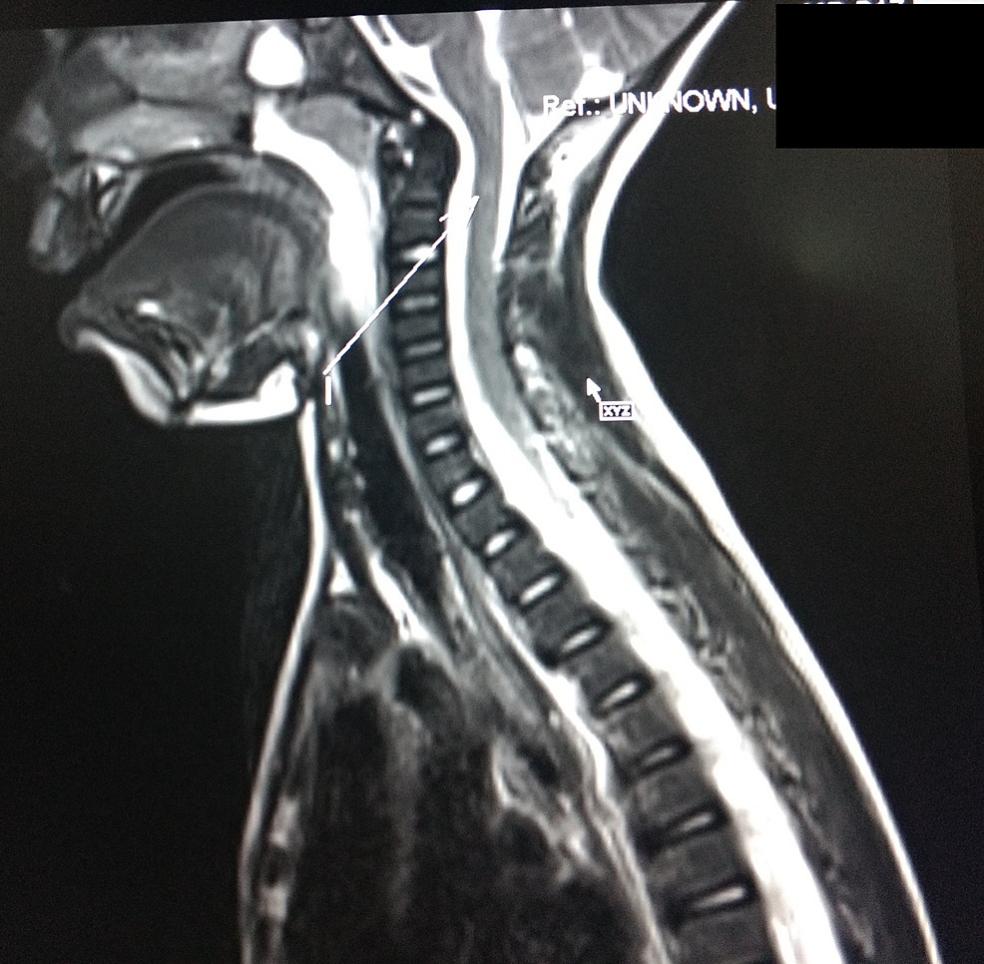
Saggital section MRI spine showing intramedullary T2W/TIRM hyperintensity in the cervical cord, notably in the upper cord at C2, C3, and upper C4 levels MRI: magnetic resonance imaging

**Figure 4:**
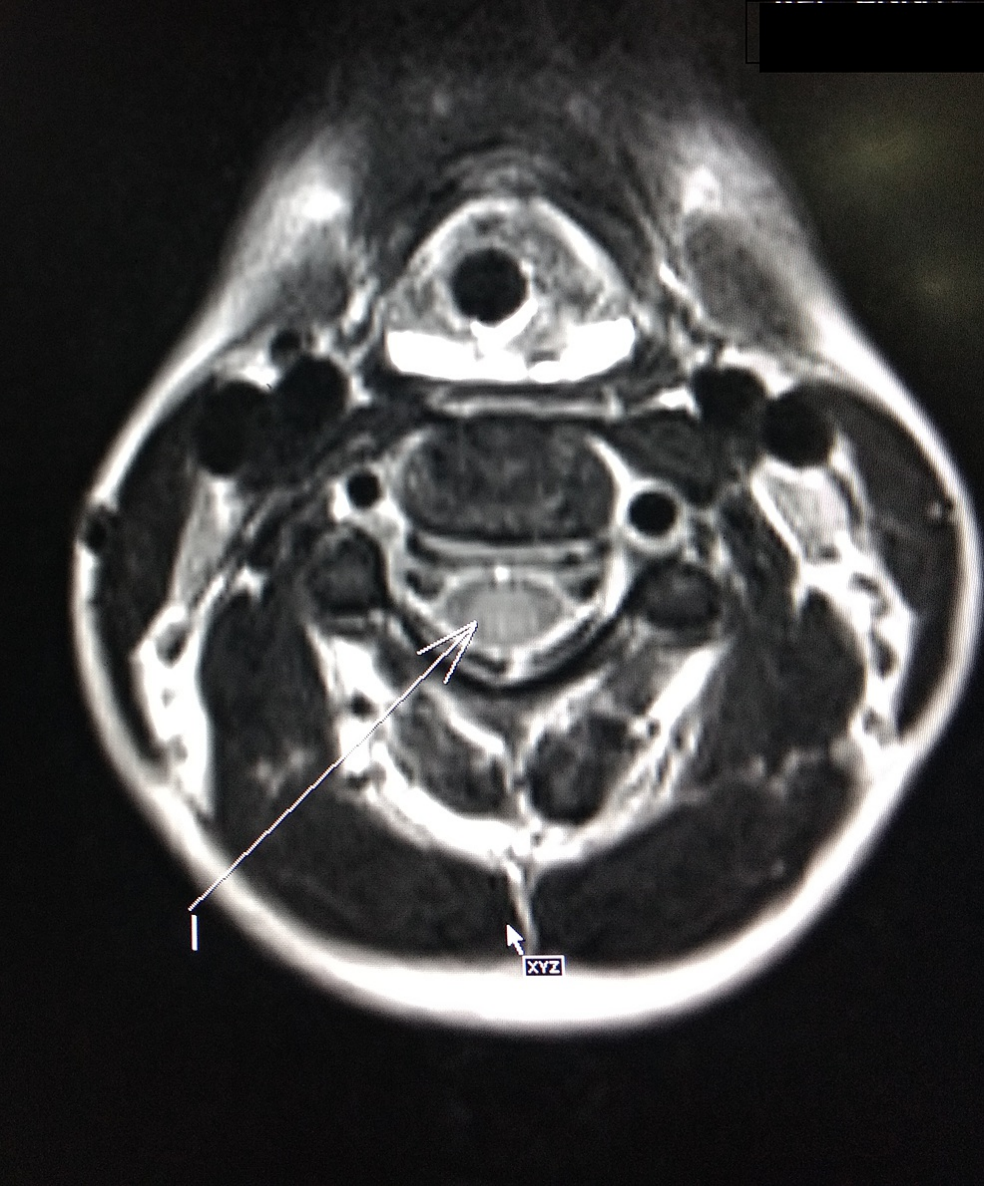
Axial section MRI cervical spine showing intramedullary T2W hyperintensity Note that on the axial image it is seen predominantly involving the central region, relatively sparing the lateral aspect MRI: magnetic resonance imaging

A four-year-old male child presented to us with weakness of voice, nasal regurgitation of food, and breathing difficulty for the past five days. No other significant past medical history was forthcoming from the parents. When the child presented to us, his higher mental functions were intact, palatal movements were absent, gag reflex was absent and so was the cough reflex. Muscle tone was decreased in all four limbs. DTRs were brisk, and the plantar reflex was absent. Bowel and bladder control was intact and there was no neck rigidity. A fundus examination was done, which was normal. CSF analysis was also normal. MRI brain was suggestive of intramedullary hyperintensities in the cervical cord, notably C2, C3, and upper C4 body levels (Figures 3, 4). NCV done on the first day of admission showed demyelination in nerves of both upper limbs and lower limbs. We treated the patient as a case of GBS variant in light of the brisk DTRs, with IVIG of 2 gm/kg over 72 hours. After the third day of IVIG, a gag reflex was observed. DTRs became normal by the fifth day of admission.

## Discussion

GBS is considered an inflammatory condition of the peripheral nervous system. It is the most common cause of acute flaccid paralysis in the world, with males more at risk compared to females. Additionally, it has been found that the risk of incidence of GBS increases with each decade of life [5]. Regardless, a large number of children in pediatric clinics would present with acute flaccid paralysis, with the typical history of weakness, with or without pain and/or sensory disturbances, starting in the lower limbs, and gradually progressing to the upper part of the body, a pattern that has been called Landry’s ascending paralysis. GBS should be considered as part of the spectrum of flaccid paralysis, rather than a single clinical entity due to the presence of heterogeneous clinical variants that often cause delays in the diagnostic process. Classically a clinically diagnosed disorder, CSF analysis and electrophysiological studies aid in the diagnosis of GBS [6]. NCV studies not only provide evidence of peripheral nervous system dysfunction but also help in classifying GBS into axonal and demyelinating subtypes, which has a role to play in prognosticating the patients' outcomes [7]. Although, an indispensable tool in the diagnosis of GBS, NCV could often be normal in the first two weeks following the beginning of the illness. We also had normal NCV findings in patients described in case 1 and case 3. Diagnosis in these cases was aided by a sharp clinical acumen owing to the awareness about the variants of GBS, complemented with suggestive MRI findings and CSF analysis showing albumin-cytological dissociation.

An important part of the clinical evaluation of GBS involves the careful exclusion of other etiologies [8]. We have described our cases very briefly in this manuscript, without giving too many details about the differential diagnoses we considered and excluded because our principal aim was to demonstrate how different variants of GBS could potentially manifest. These include weakness without sensory involvement (pure motor variant); weakness of only the cranial nerves, as in case 5; weakness limited to the upper limbs, as in case 4; cranial nerve weakness with change in the level of consciousness (Bickerstaff brainstem encephalitis), as in case 2; or the Miller Fisher variant, which, when fully manifested, consists of ophthalmoplegia, ataxia, and areflexia [9], as in our case 1. Other atypical presentations could include asymmetrical weakness of limbs, or predominantly proximal or distal, or weakness beginning in the upper limbs or simultaneous involvement of all four limbs, or weakness preceded by severe localized or diffuse pain [10]. Pain in GBS could be severe, frequently occurring as the first symptom, which has also been reported to last longer than the time it takes for complete resolution and recovery from the weakness. It is probable that involvement of the sensory fibers results in more severe pain [11,12].

Young children, particularly those less than six years of age, could present with atypical symptoms, such as refusal to bear weight, irritability, unsteady gait, diffuse pain, or meningism [13]. Some children may also have normal or even exaggerated DTRs on examination, as was seen in our case 3 and cases 2 and 5 respectively. Failure to promptly recognize variants and atypical presentations of GBS might subject a young child to not only unnecessary investigations but also add considerably to parental anxiety. Disease progression is rapid with the nadir of weakness occurring at around four weeks after the onset of illness. Cardiac arrhythmias and blood pressure fluctuations are also seen owing to the involvement of the autonomic nervous system. Of note, 20% of patients develop respiratory failure necessitating the use of mechanical ventilation [1]. GBS is thought to be an aberrant immune response to an infectious trigger that causes damage to the peripheral nervous system, and therefore the mainstay of management of GBS is IVIG and plasma exchange. Surprisingly, oral steroids and intravenous methylprednisolone are not effective in this disorder [14]. IVIG, when started within the first two weeks from the onset of illness, has been proven to be effective, and has several advantages over plasma exchange therapy. Since IVIG is easier to administer, is widely available, and has fewer side effects compared to plasma exchange, it has become the standard of care for patients with GBS; 60-80% of patients are able to walk independently six months after the onset of weakness, with or without undergoing treatment [15]. GBS is a monophasic illness, though relapses can occur in up to 5% of cases [15]. A second course of IVIG may be needed if relapses occur.

## Conclusions

GBS is a life-threatening disorder and is associated with rapid progression to the involvement of respiratory muscles. Often a delayed diagnosis, particularly in young children, it may result in emergency situations. Patients may die from ventilatory insufficiency, cardiac arrhythmias, or pulmonary complications. A working knowledge of the atypical forms of GBS is therefore important in order to recognize its variants so that early therapy can be instituted and complications avoided. IVIG therapy is effective, easy to administer, and remains the mainstay of management for GBS, in conjunction with supportive measures and meticulous monitoring for deterioration and autonomic instability.
